# Editorial: Primary and acquired resistance in lung cancer

**DOI:** 10.3389/fonc.2023.1310331

**Published:** 2023-11-01

**Authors:** Rossella Bruno, Michele Simbolo, Iacopo Petrini

**Affiliations:** ^1^ Unit of Pathological Anatomy, University Hospital of Pisa, Pisa, Italy; ^2^ Department of Diagnostics and Public Health, Section of Pathology, University of Verona, Verona, Italy; ^3^ Medical Oncology, Department of Translational Research and New Technologies in Medicine and Surgery, University of Pisa, Pisa, Italy

**Keywords:** lung cancer, targeted therapy, immunotherapy, predictive biomarkers, primary resistance, acquired resistance

Targeted therapies and immunotherapy have significantly enhanced treatment of patients with advanced and metastatic non-small cell lung cancer (NSCLC), which accounts for about 85% of all lung cancer cases (1). The molecular characterization of non-squamous tumors is necessary to identify patients harboring targetable alterations (2). Despite the continuous advances of precision medicine, primary and acquired drug resistance remains a challenge (2).

The articles featured in this Research Topic mainly focus on *EGFR* and *KRAS* addicted tumors, collectively accounting for approximately 45% of NSCLC. The authors describe resistance mechanisms to tyrosine-kinase inhibitors (TKIs) and immune-checkpoint inhibitors. Additionally, this Research Topic includes a study exploring the predictive role of genetic alterations in small cell lung cancer (SCLC).

In NSCLC, *EGFR* exhibits somatic mutations of the intracellular tyrosine-kinase domain mainly between exons 18-21. The majority of these mutations are predictive of TKI response, while a minority confers resistance to specific treatments (2). Resistance mechanisms vary depending on drugs and include on-target (secondary alterations within the same gene) and off-target mechanisms (alternative pathway activation, histological transformation). The acquisition of the T790M mutation within *EGFR* exon 20 is the most common resistance mechanism to first (gefitinib, erlotinib) and second-generation (afatinib) TKIs. Hsieh et al. reported a higher T790M mutation rate with first compared to second-generation TKIs. Tumors carrying T790M mutation respond to the third-generation EGFR inhibitor, osimertinib, which was initially approved for patients with this acquired resistance mechanism. Currently, osimertinib is the preferred drug for the first-line therapy of tumors with *EGFR* common activating mutations (3). There are not predominant resistance mutations for third-generation TKIs (4). Mahfoudhi et al. reviewed preclinical studies evaluating acquired resistance to third-generation EGFR TKIs across all treatment lines. Secondary *EGFR* mutations, like C797S, occur in approximately 10% of patients. In contrast, most tumors activate alternative pathways bypassing EGFR inhibition (i.e. *MET* amplification). Recent studies, as reported by Fabrizio et al., explored the influence of epigenetic alterations (DNA methylation and miRNA deregulation) on treatment resistance. There is an emerging evidence for a role of multi-gene and genome-wide global methylation profile, particularly when liquid biopsy is employed. In the same context, Lee et al. showed how platelet activation can confer acquired resistance to EGFR TKIs, thus suggesting new therapeutic strategies.

Mutations affecting *KRAS* gene are the most frequent in lung cancer, those specific to codon 12 are especially related to smokers. To date, only *KRAS* G12C inhibitors are approved for the second-line treatment of NSCLC patients who progress to a first-line immunotherapy or chemo-immunotherapy (2). KRAS inhibitors specific for G12D mutation are under evaluation in preliminary clinical trials (5). Therefore, the initial treatment for NSCLC patients carrying *KRAS* mutations is immunotherapy or chemo-immunotherapy, according to PD-L1 expression levels. Alternatively, chemotherapy is considered for patients with contraindication for immunotherapy. As described by Fancelli et al., different *KRAS* mutations have distinct characteristics and behaviors. Improved outcomes are observed with immunotherapy alone or in combination with chemotherapy when compared with chemotherapy alone, especially for mutations G12C, G12D and G12A, while a poorer prognosis is associated with G12V mutation. In clinical trials KRAS G12C inhibitors obtain objective responses between 30-50%, with a median progression free survival (PFS) of 5.6-6.5 months (6, 7). Resistance mechanisms to KRAS G12C inhibitors are poorly characterized. Ning et al. reviewed non-genetic mechanisms, including epithelial to mesenchymal transition, which are responsible of adaptive drug resistance and treatment failure. Considering the relative low response rate and PFS of KRAS inhibitors, identification of factors impacting response is crucial. Strategies promoting mesenchymal-to-epithelial transition, as well as the inhibition of YAP (oncoprotein acting downstream Hippo pathway) should be further investigated.

As mentioned before, immunotherapy is an important option for patients without targetable alterations (8) not only in the advanced settings, but also in early stages as neoadjuvant or adjuvant treatment (9). Neoadjuvant chemo-immunotherapy improved overall survival compared to chemotherapy in phase III clinical trials (10, 11). Alì et al. showed that neoadjuvant chemo-immunotherapy improved pathological responses compared to chemotherapy alone, and correlated with a better overall survival and event free survival in resectable NSCLC. However, a substantial percentage of patients do not respond to neoadjuvant chemo-immunotherapy. Consequently, there is an urgent need for predictive biomarkers. PD-L1, predictive of response for stage IV patients, has a limited utility in the neoadjuvant setting. In the same study authors observed that after chemo-immunotherapy residuals tumors showed enhanced expression levels of *YAP/TAZ* and *CTLA4* genes opening new interesting scenarios.

When the first-line chemo-immunotherapy or immunotherapy alone fails, chemotherapy becomes the standard second-line option, but it is often associated with toxicity and limited effectiveness. The combination of anti-angiogenetic drugs with taxane has improved patients’ outcome: for example, the addition of ramucirumab to docetaxel slightly increased overall survival after progression to a first-line chemotherapy (12). The effect of this combination after immunotherapy remains poorly characterized. Nevertheless, in preclinical studies it has been demonstrated that anti-angiogenic drugs can modulate tumor microenvironment enhancing the activity of immune check-point inhibitors (13). Kareff et al. evaluated the use of ramucirumab and docetaxel following disease progression on chemotherapy and immunotherapy combination. Their data support the advantageous effect of a combined chemotherapy and anti-angiogenic therapy after first-line immunotherapy exposure.

Precision medicine perfectly suits NSCLC, whereas therapeutic options are still limited for SCLC (15% of all cases). SCLC patients typically achieve tumor shrinkage with the first-line of chemotherapy, but, thereafter, they often rapidly experience disease progression (14). SCLC genetic landscape could help to understand how to stratify tumors improving prognosis definition. In their study Tang et al. used whole exome sequencing data to identify significant genetic differences, developing a classifier capable of predicting chemoresistance, chemosensitivity, and the risk of recurrence in SCLC.

Comprehensive genome profiling is providing valuable insights into resistance mechanisms to lung cancer treatments. However, it is important to recognize that various resistance alterations can coexist within the same tumor or between primary tumors and metastatic sites. It is essential to thoroughly evaluate resistance at molecular levels to track the genetic evolution of cancer and optimize treatment.

## Author contributions

RB: Writing – original draft, Writing – review & editing. MS: Writing – original draft, Writing – review & editing. IP: Writing – original draft, Writing – review & editing.
